# Spatial proteomics of the tumor microenvironment in melanoma: current insights and future directions

**DOI:** 10.3389/fimmu.2025.1568456

**Published:** 2025-05-15

**Authors:** Chiara Bungaro, Michele Guida, Benedetta Apollonio

**Affiliations:** Rare Tumors and Melanoma Unit, IRCCS Istituto Tumori “Giovanni Paolo II”, Bari, Italy

**Keywords:** spatial proteomics, tumor microenvironment, melanoma, immunolocalization, mapping & localization

## Abstract

Over the past years, cancer research has transitioned from a ‘cancer cell-centered’ focus to a more integrative view of tumors as dynamic ecosystems. This paradigm shift emphasizes the tumor microenvironment (TME) as a complex network of interacting cellular and acellular components, where tumor cells orchestrate a supportive environment that facilitates progression, metastasis, and immune evasion. Understanding the spatial organization of these components within the TME is crucial, as the positioning and interactions between cancerous and non-cancerous cells significantly influence tumor behavior and therapy response. Spatial proteomics has emerged as a powerful tool for TME analysis, enabling the detection and quantification of proteins within intact tissue architecture at subcellular resolution. This approach provides insights into cellular interactions, signaling pathways, and functional states, facilitating the discovery of novel biomarkers and therapeutic targets linked to specific tissue regions and cellular contexts. Translating spatial proteomics into clinical practice requires overcoming challenges related to technology refinement, standardization of workflows, and adaptation to routine pathology settings. Melanoma is an aggressive, highly immunogenic malignancy with variable response rates to existing immunotherapies. Given that over half of patients treated with immune checkpoint inhibitors (ICIs) fail to respond or experience disease progression, the identification of novel biomarkers and therapeutic targets to enhance current therapies is urgently required. Spatial imaging technologies are increasingly being utilized to dissect the complex interplay between stroma, melanoma, and immune cell types within the TME to address this need. This review examines key spatial proteomics methods, their applications in melanoma biology, and associated image analysis pipelines. We highlight the current limitations, and future directions, emphasizing the potential for clinical translation to guide personalized treatment strategies, inform prognosis, and predict therapeutic response.

## Introduction

Over the last few decades, cancer research has undergone a significant paradigm shift: from a ‘cancer-centered’ view, primarily focused on the genomic aberrations of neoplastic cells, to a more comprehensive understanding of tumors as complex ecosystems. In this new framework, tumors are seen as dynamic entities where both cellular and acellular components form an intricate network of co-evolving interactions—the tumor microenvironment (TME) (1). This holistic perspective recognizes that tumor cells act not in isolation but as central orchestrators of a tumor-supportive environment, actively recruiting and reprogramming non-immune and immune cells, remodeling the vasculature, and altering the extracellular matrix to support progression and metastasis.

Mapping the spatial location of the different cellular components is crucial as the TME is a highly organized, structured environment where the positioning of different cell types is essential for their function. For this reason, the spatial relationships between tumor cells, immune cells, stromal cells, and blood vessels are fundamental to tumor progression, immune evasion, and therapy resistance.

While tissue studies remain crucial for cancer diagnosis, patient stratification, and treatment recommendations, the techniques routinely used for these investigations (e.i. immunohistochemistry) are limited to the low number of markers that can be simultaneously visualized. In the past few years, more studies have been focused on the development of new multiplexed technologies and analysis methods aimed at preserving tissue architecture by spatially resolving the complexity of the TME, mapping different cell types, and understanding their reciprocal interactions and their function.

Among them, spatial proteomics, which allows the detection and quantification of proteins within the context of tissue architecture, has been recognized as one of the most promising methods for TME analysis (2). By mapping protein expression patterns at subcellular resolution, spatial proteomics provides insights into cellular interactions, signaling pathways, and functional states of cells within the TME. This new set of information could be used for the discovery of novel biomarkers and therapeutic targets that are tightly linked to specific tissue regions and cellular contexts. It also opens the door to identifying novel therapeutic combinations, as spatial proteomics can reveal how different treatment modalities may alter the spatial dynamics of the TME. However, while spatial proteomics offers immense potential for understanding cancer biology, its translation into clinical practice remains a challenge. It is critical to develop methods and platforms that can be easily adapted to routine pathology labs and clinical settings. This requires not only refining technologies for better sensitivity and resolution but also developing standardized workflows and protocols that can be widely adopted in clinical practice. The goal is to provide pathologists and clinicians with actionable approaches that can guide personalized treatment strategies, inform prognosis, and predict response to therapies.

Over the past few years, spatial proteomics has significantly advanced our understanding of the melanoma TME. Although melanoma is highly immunogenic and several immunotherapy-based treatments are available, a significant proportion of patients, particularly those with late-stage disease, still fail to achieve durable responses or experience disease progression (3–6). This clinical challenge underscores the urgent need to identify novel biomarkers for predicting treatment outcomes and to discover new therapeutic targets that can improve the effectiveness of current immunotherapies. To address this critical knowledge gap, spatial imaging technologies have shown critical potential.

In this review, we examine the key methods used in spatial proteomics, their applications in melanoma biology, and the image processing and analysis pipelines associated with these technologies. We also address the current limitations and outline future directions for advancing spatial proteomics.

## Spatial proteomics methods

**Figure 1:**
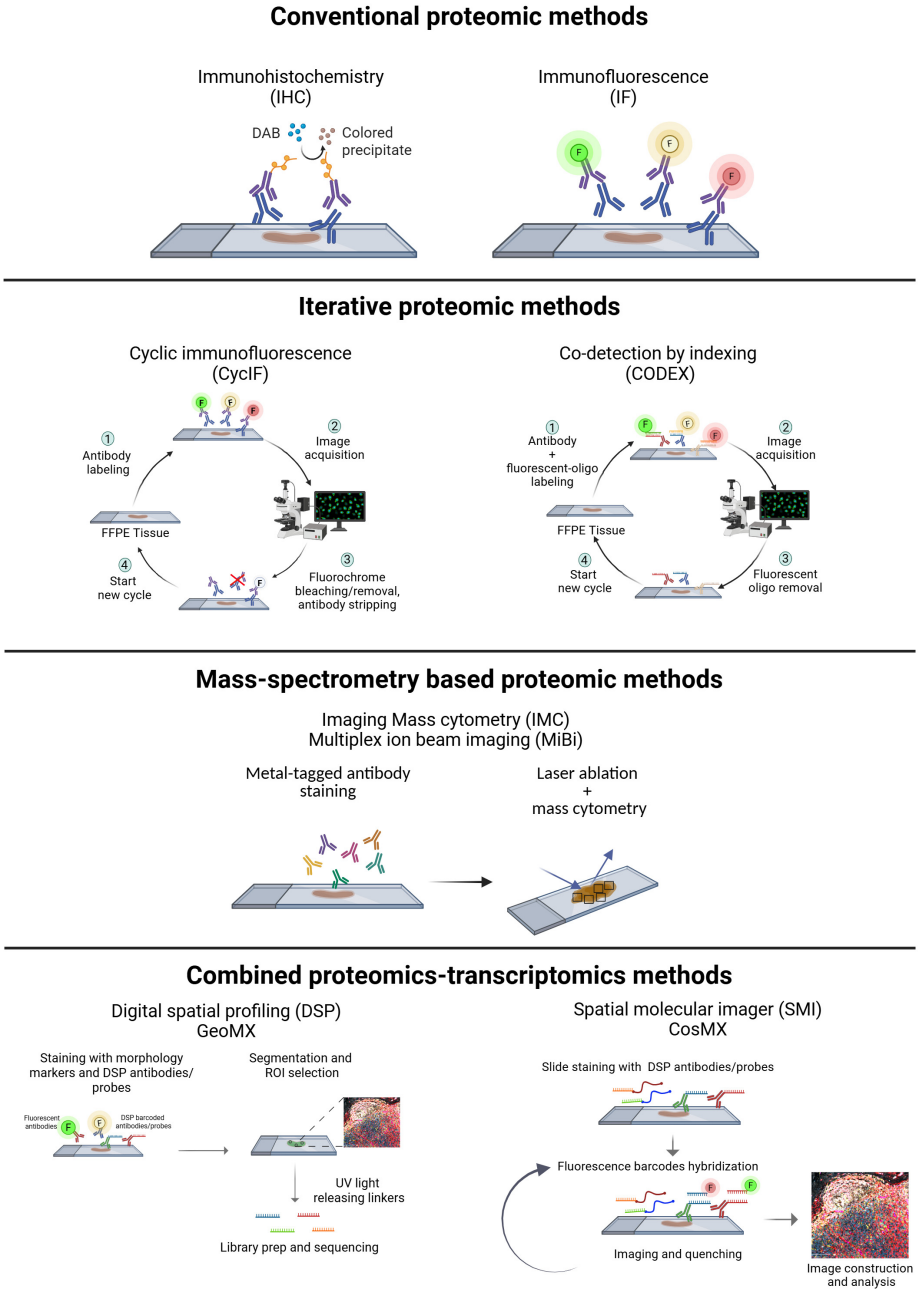
Experimental workflow of the available spatial proteomics technologies: conventional [IHC (immunohistochemistry), IF (immunofluorescence)], iterative [CyCIF/HIFI (cyclic immunofluorescence, hyperplexed immunofluorescence imaging), CODEX (Co-detection by indexing)], mass spectrometry based [IMC (imaging mass cytometry), MiBi (multiplex ion beam imaging)], and methods that combine proteomics and transcriptomics [DSP (digital spatial profiling-GeoMX) and SMI (spatial molecular imager-CosMX)]. Created in https://BioRender.com.

While the core principle of antibody-staining remains consistent across most spatial proteomic approaches, they differ in detection methods. Different moieties attached to the (primary/secondary) antibodies used for protein target detection act as signal amplifiers or identifiers, and are based on enzymes, fluorescence, or mass spectrometry (**Figure 1**, **Table 1**).

**Table 1:** Summary of methods applied in spatial proteomics.

Spatial Proteomic Method	Number of targets	Antibody detection method	Image acquisition method	Whole slide	Sample type	Resolution	Pros	Cons
IHC	Up to 3	Chromogenic amplification	Optical	Yes	FFPE	Subcellular	High sensitivity, reproducibility	Low throughput, no direct correlation between protein expression and signal intensity
MxIF	Up to 6	Conjugated fluorochrome	Fluorescence	Yes	FFPE FF	Subcellular	High sensitivity	Low throughput
CyCIF/HiFI	>40	Conjugated fluorochrome	Fluorescence	Yes	FFPE FF	Subcellular	Multiple markers	Antibody stripping optimization, need for specific instruments (if automated), time consuming (if manual)
CODEX ImmunoSABER	Up to 100	DNA barcode	Fluorescence	Yes	FFPE FF	Subcellular	High throughput	Antibody optimization and availability
MiBI IMC	>40	Metal	Mass Spectrometry	No	FFPE	0.4-0.7μm 1μm	High throughput, no autofluorescence	Long acquisition time, no amplification system, tissue disruption
DSP (GeoMX)	Up to 3 (fluorescence) >100 barcode	Fluorochrome-tagged + oligo-tagged	Fluorescence	No	FFPE FF	Subcellular	High throughput, coupled to RNA analysis	Limited markers selection, difficult single cell analysis
SMI (CosMX)	>100	Fluorochrome-tagged probes	Fluorescence	No	FFPE FF	Subcellular	High throughput, coupled to RNA analysis, single cell detection	Costs, low scalability

IHC, immunohistochemistry; MxIF, multiplex immunofluorescence; CyCIF, cyclic immunofluorescence; HiFI, hyperplexed immunofluoresce imaging; CODEX, co-detection by indexing; MiBI, multiplexed ion beam imaging; IMC, imaging mass cytometry; DSP, digital spatial transcriptomic; SMI, spatial molecular imager; FFPE, Formalin-Fix Paraffin Embedded; FF, fresh frozen.

Conventional immunohistochemistry (IHC) is widely used in routine pathology for tumor diagnosis and classification. It is based on the simultaneous use of up to two antibodies directed against specific markers, and an enzyme-based detection through horseradish peroxidase or alkaline phosphatase (7). This approach has high sensitivity and is well-established, but it is limited in the number of proteins simultaneously detected. To overcome this limitation multiplex IHC (mIHC) protocols, based on multiplexing sequential staining strategies, have been optimized. mIHC can be based on two main approaches: (i) same-slide iterative labeling, digital scanning, and antibody stripping; (ii) sequential tissue slices staining with one/two antibodies simultaneously (8), digital scanning, and images overlapping (9).

These techniques expand the number of detected markers but, as chromogenic amplification provides a non-linear correlation with protein expression levels, they lack a direct correlation between protein expression level and signal intensity. Additionally, the use of sequential tissue slice staining introduces challenges in maintaining cell identity and consistency across slices.

Multiplex immunofluorescence (MxIF) allows the simultaneous detection of different protein targets at the cellular level. Fluorescence spectral overlap limits detection to a maximum of 4 or 5 markers, or up to 6 if using maximum laser number coupled with deconvolution algorithms.

The number of detectable markers can be increased using Cyclic Immunofluorescence (CyCIF), which is based on the same antibody staining cycle principle employed in mIHC (10). Signal removal between staining cycles can be achieved by either stripping the antibody or its label, or through fluorophore photobleaching, such as Iterative Bleaching Extends Multiplexity (IBEX) (11). While these methods enable the detection of a greater number of markers compared to conventional immunofluorescence (IF), they require careful optimization of the antibody staining sequence. Additionally, they often depend on specialized automated systems that minimize handling time and improve efficiency, although at high costs.

Recent studies have introduced a new pipeline for cyCIF, called hyperplexed immunofluorescence imaging (HIFI), which employs a manual, cost-effective, and instrument-free approach (12, 13). This method enables high-throughput data acquisition using standard benchtop reagents and conventional slide-scanning microscopes, facilitating the democratization of high-throughput spatial proteomics. However, despite their accessibility and whole-slide imaging capabilities, manual HIFI methods are time-consuming compared to platform-dependent proteomic techniques. They require continuous operator presence, optimization of antibody panels (to avoid antigen damage during elution), and protocol durations that vary with the number of staining rounds. Additionally, the analysis pipelines for these methods are not yet fully standardized.

Co-detection by indexing (CODEX) is another cyclic imaging technique that involves staining with antibodies conjugated to oligonucleotide tags with 5’ overhangs of varying lengths. This is followed by multiple imaging cycles, enabling the detection of up to 100 markers (14). The signal from DNA-labeled antibodies can be further amplified using sequential hybridization reactions, such as Immuno-SABER. While these methods enable maximal multiplexing and offer a powerful tool for spatial proteomics, they come with certain limitations. One of the challenges is the need for additional antibody validation after the conjugation step to ensure that the conjugated oligonucleotide tags do not interfere with antibody binding. Moreover, the absence of an amplification system for the antibody tags themselves means that lowly or diffusely expressed markers are difficult to detect, as the signal from these markers may not be strong enough to distinguish from background noise.

Currently, CODEX-based imaging has been used in numerous studies to achieve high-plex imaging of whole tissue samples. However, the costs associated with this technology present a challenge to its widespread application. Other multiplex approaches are based on the use of metal-tagged antibodies, which provide unmatched protein identification capabilities and can quantify more than 40 proteins simultaneously. They are based on Time-of-flight (TOF) mass spectrometry and differ in how metals are extracted from the tissue: using either secondary ionization [MiBi-Multiplexed ion beam imaging (15)] or laser ablation [IMC-imaging mass cytometry (16)].

While these approaches do not require multiple tissue slices or cycles of antibody staining/stripping, they do not have an amplification system and require long time for image acquisition. Moreover, secondary ionization and laser ablation cause tissue destruction, and samples cannot be used for downstream applications (e.g. digital spatial profiling).

Other methods combine the simultaneous detection of spatial proteome and transcriptome to achieve a more detailed tissue resolution. The Digital Spatial Profiler (DSP) GeoMX employs fluorochrome-tagged antibodies (morphology markers) for cell identification, alongside DSP antibody tagged with unique barcoded photocleavable oligonucleotide linkers. Fluorescence-based imaging is used for the segmentation of the cell types of interest with morphology markers. Tissues are then exposed to UV light which releases the photocleavable linkers. Subsequent library construction and sequencing allow for digital readout and spatial mapping of the protein targets. Notably, DSP also includes RNA probes, offering *in situ* transcriptomic mapping (17). Compared to other spatial proteomics approaches, DSP increases the number and variety of spatially resolved targets, however it does not achieve single-cell resolution.

In contrast, the Spatial molecular imager (SMI) CosMx utilizes a high-plex *in situ* fluorescence-based imaging approach for the concurrent detection of both RNA and protein. Tissues are incubated with oligonucleotide-tagged antibodies (for proteins) and probes (for RNA). Fluorescent reporters with unique barcodes are sequentially hybridized and imaged, with the signal quenched after each round. This cyclic process builds a unique fluorescent signature for each protein and RNA target, allowing for its identification and quantification at a precise location (18).

Despite its high throughput and resolution capabilities, CosMX experiments can be considerably expensive, potentially restricting its accessibility for some research groups or larger-scale studies.

## Application of spatial proteomics to melanoma tumor microenvironment

Characterizing the melanoma TME, its cellular composition, and the spatial relationships between its cellular components is increasingly vital, especially in the era of immunotherapy. Understanding cell phenotypes, their precise location, and interactions within the TME can provide critical insights into how tumors evade immune surveillance and respond to treatment (**Table 2**).

**Table 2:** Application of spatial proteomics for the study of melanoma TME.

Reference	Model	Technology	Sample type	Multiplexed targets	Sample size	Clinical findings
Gide TN et al (19)	Human	MxIF	Primary melanoma pre/post treatment	4	~40	Patients responding to ICIs have increase numbers of intratumoral and peritumoral CD8^+^ T cells together with higher numbers of PD-L1^+^ cells
Giraldo NA et al (22)	Human	MxIF	Melanoma metastasis	6	93	Long-term survivor patients: "inflamed" TME. Tumor cells near high densities of CD8^+^ or PD-1^+^ cells. Non-survivors: high densities of CD163^+^ cells lacking PD-L1, often close to other macrophages.
Mendoza-Valderrey A et al (23)	Human	IHC MxIF DSP	Melanoma metastasis (brain)	IHC (1) MxIF (3) DSP (56)	52	Higher infiltration of CD3^+^ and CD20^+^ lymphocytes in tumor brain metastasis associated with longer overall survival
Bosisio FM et al (24)	Human	CycIF	Primary melanoma	39	29	Cellular neighborhoods associated to traditional pathological classifications (e.g. brisk/non-brisk immune infiltrate, early/late regression)
Nirmal AJ et al (25)	Human	CycIF	Primary melanoma	30	11	Histological progression associated with formation of myeloid niches and T cell exhaustion
Liu D et al (26)	Human	CycIF	Primary melanoma, melanoma metastasis	23	37 longitudinal samples	Increase of NGFR^hi^ tumor cells and decreased numbers of CD8^+^, CD4^+^ FoxP3^-^ lymphocytes over time (during progression, ICIs treatment and resistance)
Hickey JW (27)	Mouse, Human	CODEX	B16F10 melanoma model Melanoma metastasis	42 mouse 58 human	Human: 6 before/after ICIs	Responder patients show higher PD-1^+^ CD8 T cells and TCF1/7^+^ CD8 T cells pre and post ICIs
Liu H (28)	Human	CODEX	Acral melanoma	22	12	Increased spatial enrichment of APOE^+^ CD163^+^ macrophages associated to invasive acral melanoma (worse prognosis)
Surwase SS et al (29)	Murine	CODEX	B16F10 melanoma model	28	–	Increased intratumoral immune activity after immunotherapy delivery with nanoparticles
Martinez-Morrilla S et al (30)	Human	IMC	Melanoma	25	60	High β2-microgluobulin, MHC-I, and LAG3 associated to improved progression-free survival and overall survival in ICIs-treated melanoma patients
Xiao X et al (31)	Human	IMC	Melanoma	35	26	Immune-hot TMEs formed by B lymphocytes and CD8^+^ and CD4^+^ T lymphocytes correlate to response to ICIs and better overall survival. Immune-cold TMEs are formed by myeloid cells in close contact with CD8^+^ T cells and are predictive of poor clinical outcomes
Moldoveanu D et al (32)	Human	IMC	Melanoma	35	67	Proliferating antigen-experienced cytotoxic T cells (CD8^+^CD45RO^+^Ki67^+^) close to melanoma cells associated with response to ICIs
Hoch T et al (33)	Human	IMC	Melanoma	41	69	CXCL9 and CXCL10 localized in patches associated with dysfunctional T cells, while CXCL13 strongly associated with B cell patches and follicles, indicating that chemokines are associated to different cellular milieu
Toki MI et al (34)	Human	DSP	Melanoma	3 fluorescence 44 DSP	60	PD-L1 expression in macrophages associated with better progression free survival and overall survival
Martinez-Morilla S et al (35)	Human	DSP	Melanoma	3 fluorescence 77 DSP	53	High stroma expression of CD95 associated with resistance to ICIs treatment
Barras D et al (36)	Human	DSP	Melanoma	4 fluorescence 79 DSP	13	Patients responding to adoptive cellular therapy with tumor-infiltrating lymphocytes (TIL-ACT) exhibit CD8^+^ TILs with increased cytotoxicity, exhaustion, and co-stimulation markers
Cabrita R et al (37)	Human	DSP	Metastatic melanoma	4 fluorescence 60 DSP	55	Co-occurrence of CD8^+^ T cells and CD20^+^ B cells in the TME is associated with improved survival and tertiary lymphoid structures formation
Helmink BA et al (38)	Human	DSP	Melanoma	4 fluorescence 22 DSP	5	TLSs are associated with markers of T cell activation and response and B cell proliferation
Beasley GM et al (39)	Human	DSP	Melanoma sentinel lymph node	3 fluorescence 59 DSP	4	High expression of dendritic cell (DC) activation markers (CD86, HLA-DR, OX40L) within the SLN tumor associated with greater overall survival
Therien AD et al (40)	Human	DSP	Melanoma sentinel lymph node	3 fluorescence 68 DSP	24	Activation markers, including Ki67-associated to B cells follicles, are increased in metastatic sentinel lymph nodes

Gide et al. (19) used MxIF to quantify densities and spatial locations of T cells and PD-L1 in the TME of melanoma patients treated with immunotherapy (both single agent and combination ICIs). They used the Opal technology coupled to the Vectra 3.0 slide scanner to obtain a 5 colors whole slide image of FFPE primary tumors and to identify area of interest (tumoral and peritumoral) that were then imaged at higher resolution (20x), and analyzed using the scanner proprietary software. They found that patients responding to ICIs had increased numbers of intratumoral and peritumoral CD8^+^ T cells together with higher numbers of PD-L1^+^ cells, both before therapy and in tumor biopsies collected early after therapy, thus confirming data obtained with IHC (20, 21). They also observed an increase of CD8^+^ expressing granzyme B, EOMES (Eomesodermin), and TBET (T-box expressed in T cells) in responder patients at early stages of treatment, indicating that effector T cells are needed for an optimal anti-tumor immune response.

However, there were some limitations in the methodology that may have restricted the depth of analysis. The study did not use multiplex immunofluorescence to differentiate between cells expressing multiple markers. Instead, authors quantified cells expressing a single marker, potentially overlooking important cell subpopulations, such as exhausted T cells which could have been characterized by co-expression of inhibitory receptors (e.g., PD-1, TIM-3). Therefore, the lack of multi-marker analysis limited the potential for identifying more immune cell phenotypes. Furthermore, the study did not incorporate spatial analysis of the immune microenvironment.

A 6plex MxIF was used to define the immune landscape of melanoma metastasis (22). Interestingly, immune cell neighborhoods were defined using an unsupervised flow cytometry-like workflow which identified spatial immune signatures associated with prognosis.

In brain melanoma metastasis, MxIF was used to confirm digital spatial transcriptomic data and to map the neural-immune architecture of the TME. The data confirmed that patients with tumor brain metastasis infiltrated with higher number of CD3^+^ and CD20^+^ lymphocytes (presumably corresponding to tertiary lymphoid structures) experienced longer overall survival, suggesting that more organized immune infiltrates can foster active anti-tumor immune responses or restrict tumor expansion (23).

These studies only partially leveraged the potential of MxIF, limiting the number of markers analyzed and thus hindering the exploration of cellular heterogeneity within the TME.

CycIF with the MILAN (multiple iterative labelling by antibody neodeposition) method (which uses a combination of a detergent and a reducing agent to remove antibodies) has been used to dissect TME cell phenotypes in a small cohort of primary cutaneous melanoma (24). Authors identified 47 functional cell populations (corresponding to tumor, epithelial, and immune cells) and different cellular neighborhoods characterized by interactions between activated and/or exhausted immune cells. These interactions were linked to traditional pathological classifications (e.g. brisk/non-brisk immune infiltrate, early/late regression), offering functional insights into classical pathological features commonly used in melanoma staging.

A recent study employing 20–30 plex CyCIF provided a more detailed characterization of primary melanoma, examining its cellular composition and structural organization (25). Through spatial analysis, the authors identified the presence of recurrent cellular neighborhoods (RCNs), spatial clusters of different cell types that change during disease progression. Specifically, the study uncovered how initial anti-tumor immune responses were progressively hindered as myeloid niches formed, leading to T cell exhaustion and eventually immune suppression. As melanoma acquires invasive properties, these changes in cellular microenvironments facilitate tumor progression. Furthermore, by integrating spatial proteomics with spatial transcriptomics, the researchers were able to identify distinct molecular programs tied to disease progression, thereby advancing our understanding of melanoma biology. This integrative approach also uncovered potential therapeutic targets that could be leveraged for future immunotherapy strategies.

CyCIF has also been used to assess melanoma evolution in longitudinal samples collected across 9 years from a single patient initially responding to ICIs and subsequently experiencing late recurrence and death (26). These studies allowed the spatial characterization of tumor-immune interactions occurring during response vs late ICIs resistance and at different metastatic sites, allowing a deeper understanding of the evolution of resistance and tumor microenvironmental heterogeneity, offering a rationale to improve combination therapies and to identify new targets.

Additional studies have utilized CODEX to investigate TME evolution during adoptive T cell therapy in mouse models of melanoma. A 42-plex antibody panel targeting immune, tumor, and stromal cells, along with functional markers (primarily checkpoints), was employed to examine immune cell infiltration and tumor inflammation dynamics in a syngeneic B16F10 model of antigen-specific T cell therapy (27). The authors reconstructed the timeline of the anti-tumor immune response, visualizing different cell neighborhoods at various stages of inflammation and tumor attack. Their findings revealed that therapeutic T cells not only target tumor cells but can also induce a shift in tumor cell phenotypes, converting them into an inflamed, anti-proliferative state. Moreover, T cells were shown to mediate the formation of both productive and unproductive tumor-immune neighborhoods, which affect therapy responses. A similar evolution of cellular neighborhoods was observed in human melanoma samples stained with a 58-plex panel, comparing responder vs non-responder patients to ICIs. Results show that greater abundance of PD-1^+^ CD8 T cells and TCF1/7^+^ CD8 T cells pre and post ICIs was associated with response to treatment. Responders also showed spatial reorganization of the TME after ICIs treatment, with the formation of tumor-immune neighborhoods highly enriched in Immune Infiltrate cellular neighbourhoods (27). These data underscore the importance of considering T cells influence on the structural reprogramming of the TME, as this process can significantly impact the magnitude and effectiveness of anti-tumor immune responses and tumor eradication. Therefore, these findings suggest that immunotherapy strategies should incorporate factors capable of restructuring the TME to enhance therapeutic outcomes.

More recently, CODEX was employed in a multi-omics study to define and functionally assess the transition from *in situ* to invasive acral melanoma. By integrating genomic sequencing with various transcriptomic approaches and a 22-plex CODEX panel, the authors identified molecular tumor subtypes characterized by increased epithelial-mesenchymal transition and spatial enrichment of APOE^+^ CD163^+^ macrophages as markers of invasive acral melanoma, with a worse prognosis (28). A recent study has also shown the applicability of the CODEX technology to study TME changes in animal models treated with novel combination therapies (29).

In addition to CODEX, IMC has also been used to stratify patient responses to immunotherapy. In a study using a 26-plex panel on 60 melanoma samples from ICI-treated patients, the application of the AQUA software, which calculates the cumulative signal intensity per unit compartment area, identified beta-2-microglobulin expression as a predictor of ICI response (30). In addition, Xiao and colleagues used a 35-plex and identified 6 different patient archetypes (spatial cellular neighborhoods) predictive of anti-PD-1 responses. In line with other spatial proteomics studies (25, 27), they observed that immune-hot TMEs are formed by CD8^+^ T cells surrounded by CD4^+^ and B lymphocytes, and correlate to response to ICIs and better overall survival. On the other hand, immune-cold archetypes are characterized by myeloid cells in close contact with CD8^+^ T cells and are predictive of poor clinical outcomes (31). Another IMC study quantifying the expression of 35 protein markers in 67 pre-treatment melanomas, demonstrated that the abundance of proliferating antigen-experienced cytotoxic T cells (CD8^+^CD45RO^+^Ki67^+^) and their proximity to melanoma cells were associated with positive response to ICIs (32).

Other IMC-based studies have employed modified protocols to simultaneously detect protein markers and mRNA targets for chemokines, enabling a more comprehensive analysis of T lymphocytes activation and/or dysfunction and their patterns of interaction in the TME (33).

Digital Spatial Profiling (DSP) GeoMX has been applied in melanoma biology to discover predictive markers for immunotherapy response in metastatic patients. Two independent studies identified PD-L1 expression in macrophages, but not tumor cells, as the strongest predictor of response to ICIs, while CD95 expression in immune cells was associated with immunotherapy resistance (34, 35). In a phase I study of metastatic melanoma patients treated with adoptive cellular therapy with tumor-infiltrating lymphocytes (TIL-ACT), DSP analysis revealed distinct immune profiles in responders. At baseline, responder patients exhibited CD8^+^ TILs with increased cytotoxicity, exhaustion, and costimulation markers, while myeloid cells showed elevated type I interferon signaling (36). DSP has also been used to identify and characterize tertiary lymphoid structures (TLS) within the melanoma TME. Two separate studies revealed differential expression of various activation and response markers in T lymphocytes residing within TLS compared to those outside, which exhibited a dysfunctional phenotype (37, 38).

DSP studies in melanoma have also been extended to the characterization of sentinel lymph nodes (SLNs). Beasley et al. demonstrated an association between dendritic cell (DC) activation markers (CD86, HLA-DR, OX40L) within the SLN tumor and overall survival (OS), with lowest expression in patients with OS < 1 year and highest in those with OS > 8 years (39). Another study utilizing a 68-antibody DSP panel to analyze B cell follicles in melanoma SLNs revealed significantly higher expression of multiple activation markers, including Ki-67, within B cell regions of metastatic SLNs compared to non-metastatic SLNs. These findings suggest that B cell follicles within SLNs could be involved in orchestrating effective adaptive immune responses in melanoma even at early stages of lymph node involvement, characterized by low tumor cell infiltration (40).

In summary, spatial proteomics has significantly advanced our understanding of the melanoma TME, shedding light on its heterogeneity in relation to prognosis, its evolution during immunotherapy response or resistance, and its distinct organization across different metastatic sites. However, additional work is required to translate these insights into clinical applications and diagnostic tools.

## Image analysis workflow for spatial proteomics

**Figure 2:**
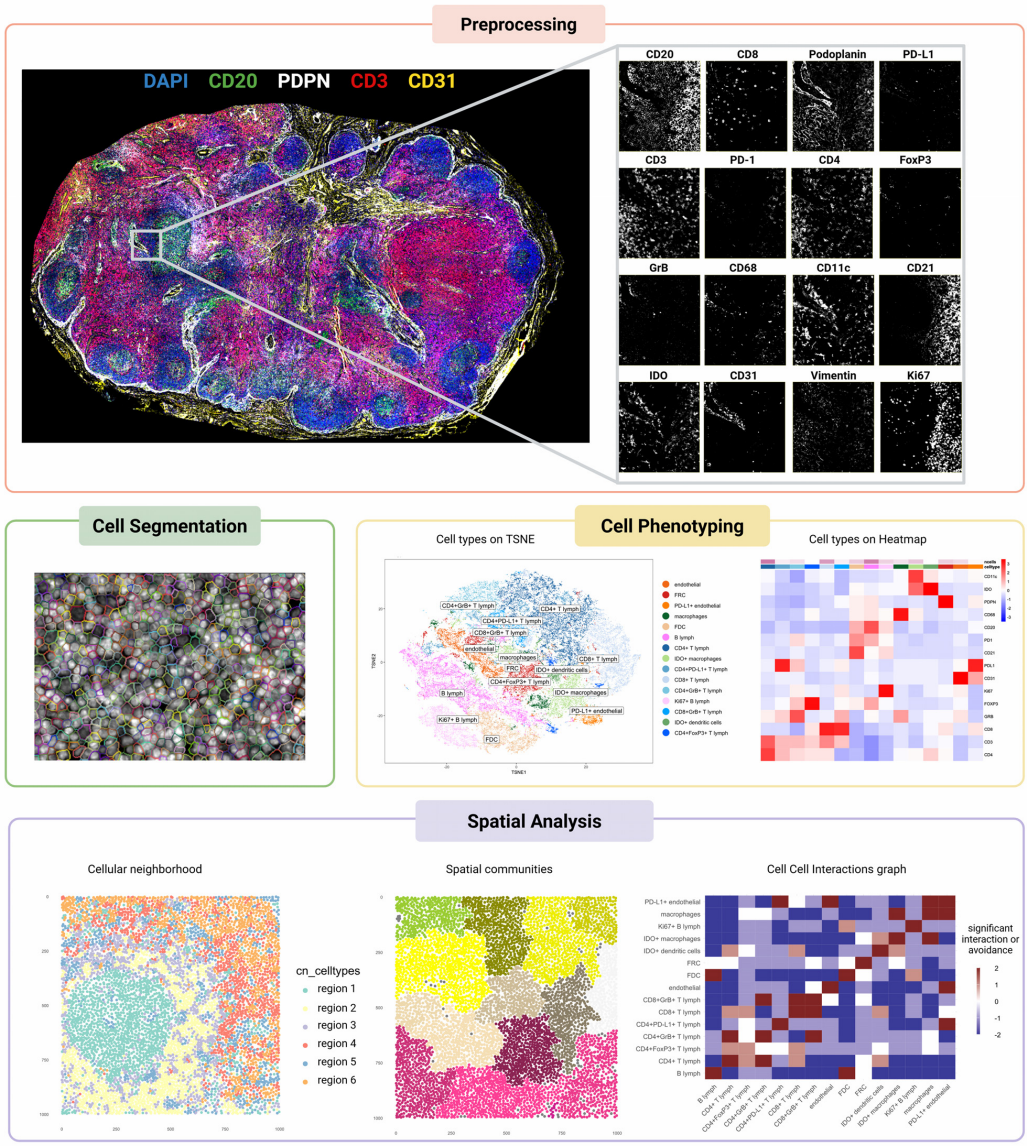
Workflow of spatial proteomics analysis applied to a multiplexed image of a metastasis-free melanoma sentinel lymph node. The pipeline includes key analytical steps, each illustrated with representative outputs. Preprocessing: image registration using the VALIS algorithm ensures alignment across imaging channels. Cell segmentation: nuclei and cytoplasmic boundaries are segmented using Cellpose. Cell phenotyping: t-SNE plot and heatmap display the results of phenotypic clustering performed with PhenoGraph. Spatial analysis: cellular neighborhood analysis, spatial community detection, and cell–cell interaction inference were carried out using the imcRtools package. Created in https://BioRender.com.

Extracting meaningful biological insights from the complex amount of data obtained with the different spatial proteomics methods requires a well-defined analytical workflow which involves four key pillars: (i) image pre-processing to correct variation in image quality, (ii) cell segmentation to identify individual cells, (iii) cell phenotyping to classify cells and reveal their functional states, and (iv) spatial neighborhood analysis to delve into the intricate communication networks between cell populations. To streamline this complex workflow and empower researchers, a series of software tools have been developed (**Figure 2**, **Table 3**).

**Table 3:** Tools and software commonly used in spatial proteomics analysis workflow.

Image Analysis Step	Specific Task	Software/Tools	Ref	Links
PREPROCESSING	Stitching	Ashlar; RAPID	(41, 47)	https://github.com/labsyspharm/ashlar https://github.com/nolanlab/RAPID
	Registration	HiFiAlignmentTool; Ashlar; Valis; RAPID;	(13, 41, 42, 47)	https://github.com/jhausserlab/HiFiAlignmentTool https://github.com/labsyspharm/ashlar https://github.com/MathOnco/valis https://github.com/nolanlab/RAPID
	Artifacts Removal	Qual-IF-AI,	(43)	https://github.com/TCWO/QualIFAI
	Background and Illumination correction	BaSiC	(45)	https://github.com/marrlab/BaSiC
	Hot Pixel corrections	IMC-Denoise	(44)	https://github.com/PENGLU-WashU/IMC_Denoise
SEGMENTATION	Machine Learning Approaches	IMC segmentation pipeline (Ilastik+ CellProfiler)	(16, 50, 51)	https://github.com/BodenmillerGroup/ImcSegmentationPipeline https://github.com/ilastik/ilastik https://github.com/CellProfiler
	Deep Learning Approaches	STARDIST; MESMER; CELLPOSE.	(52–54)	https://github.com/stardist/stardist https://github.com/vanvalenlab/intro-to-deepcell/tree/master/pretrained_models https://github.com/MouseLand/cellpose
CELL PHENOTYPING	Clustering	Phenograph; FlowSOM.	(56, 58)	https://github.com/i-cyto/Rphenograph https://github.com/saeyslab/FlowSOM
	Machine Learning Approaches	Garnett; CELESTA; Astir.	(61–63)	https://github.com/cole-trapnell-lab/garnett https://github.com/plevritis-lab/CELESTA https://github.com/camlab-bioml/astir
	Deep Learning Approaches	DeepCellTypes; CellSighter; STELLAR.	(64–66)	https://github.com/vanvalenlab/deepcell-types https://github.com/KerenLab/CellSighter https://github.com/snap-stanford/stellar
SPATIAL ANALYSIS	Neighborhood analysis	imcRtools; Histocat.	(16, 68)	https://github.com/BodenmillerGroup/imcRtools https://github.com/SchapiroLabor/histoCAT
	Cellular communities	imcRtools; Squidpy.	(16, 71)	https://github.com/BodenmillerGroup/imcRtools https://github.com/scverse/squidpy
	Interaction analysis	imcRtools; Giotto.	(16, 74)	https://github.com/BodenmillerGroup/imcRtools https://github.com/drieslab/Giotto
	Ligand-receptor signaling networks	CellChat; SpaOTsc.	(76, 77)	https://github.com/sqjin/CellChat https://github.com/zcang/SpaOTsc

### Preprocessing

Multiplex imaging techniques produce rich datasets that require rigorous preprocessing to ensure data quality, consistency, and accurate downstream analysis. This step involves addressing artifacts, aligning multi-tile or multi-round images, and optimizing overall image quality to overcome the technical challenges inherent in spatial proteomic methods. For tiled imaging techniques, stitching and registration play a crucial role in ensuring accurate and comprehensive datasets. Stitching assembles individual image tiles into a cohesive dataset, while registration aligns images across different cycles for the same tissue slide and between sequential slides to a common spatial framework. Tools like ASHLAR (Alignment by Simultaneous Harmonization of Layer/Adjacency Registration) (41) excel in both tasks by leveraging iterative optimization algorithms to achieve subpixel accuracy. Designed specifically for cyclic imaging workflows, ASHLAR addresses common issues like misalignment between imaging cycles or shifts in tissue position, ensuring a seamless composite that faithfully represents the original tissue. Similarly, VALIS (Virtual Alignment of Pathology Image Series) (42) offers a flexible and scalable approach to aligning multi-gigapixel whole-slide images and provides a modality-agnostic solution, supporting both immunofluorescence and brightfield datasets. Its unique groupwise registration method is particularly beneficial for datasets with high variability in staining or tissue distortion, as VALIS can integrate both rigid and non-rigid transformations to accommodate stretching, folding, or other deformations.

Beyond alignment, preprocessing tackles artifacts that compromise image quality. These include folding, air bubbles, dust, lint, out-of-focus areas, and uneven illumination, all of which can distort quantitative results. Automated tools such as QUAL-IF-AI (Quality Control of ImmunoFluorescence Images using Artificial Intelligence) (43) leverage deep learning to detect and correct these issues efficiently, offering a reproducible alternative to labor-intensive manual corrections​​. For IMC datasets, hot pixels—high intensity signals uncorrelated to any biological structures — are removed using intensity thresholding or spatial filters (median, Gaussian filter) or some specific pipeline such as IMC-Denoise (44).

In immunofluorescence workflows, background subtraction and illumination correction are essential to minimize signal interference and standardize intensity across the image. Techniques such as BaSiC algorithm (45) provide robust solutions for correcting uneven illumination patterns, improving the consistency of fluorescence signals across tiles and within imaging cycles​. Autofluorescence, a persistent challenge in older or archival tissues, can be mitigated using spectral unmixing or model-based approaches.

Ultimately, preprocessing pipelines like MCMICRO (Multiple-choice microscopy pipeline) (46) or RAPID (a Real-time, GPU-Accelerated Parallelized Image processing software for large-scale multiplexed fluorescence microscopy Data) (47) are indispensable for high-throughput multiplex imaging. They streamline workflows by automating quality control, stitching, and registration, while facilitating reproducibility and scalability. For instance, the MCMICRO pipeline has been successfully applied to investigate the spatial landscape of progression and immunoediting in primary melanoma (25). By addressing both general and modality-specific challenges, preprocessing enables accurate feature extraction and sets the stage for robust spatial and molecular analyses.

### Segmentation

Following image processing and alignment, cell segmentation emerges as a crucial step in analyzing cellular features. During segmentation, individual cell boundaries are computationally identified, generating binary masks that represent single cells within the image. The accuracy of cell segmentation significantly impacts the quantification of multicellular properties, such as protein expression and cell morphology. An ideal cell segmentation algorithm should effectively segment cells of different sizes and shapes within various tissue types, regardless of cell density. Additionally, it should accurately delineate both the membrane and internal compartments like the nucleus and cytoplasm. Techniques like watershed segmentation (48), are effective for isolating individual cells, however they show some limitations in accurately segmenting overlapping cells or cells with complex morphologies, which can lead to cell’s over-segmentation (49). Therefore, more advanced segmentation algorithms, such as machine learning and deep learning models, are often necessary to achieve accurate cell segmentation in complex tissues.

Machine learning-based approaches, such as random forest classifiers, have gained traction for segmentation tasks. A common workflow, IMCSegmentation Pipeline (16), involves tools like Ilastik (50), which enables pixel-based classification to distinguish between nuclei, membranes, and background regions, generating probability maps. These maps can then be processed in CellProfiler (51) to produce segmentation masks. However, these methods require extensive manual effort and parameter optimization, and their accuracy relies heavily on the quality and volume of the training dataset.

In contrast, deep learning-based models represent a transformative advance, offering superior accuracy and robustness with minimal user intervention. Models like StarDist (52), Mesmer (53), and Cellpose (54) have redefined cell segmentation, particularly in challenging tissue contexts. StarDist leverages a star-convex polygonal representation (55) to segment individual cells, particularly excelling in identifying nuclei of various shapes and sizes. Its unique approach models each nucleus as a star-shaped object, which enables robust segmentation even in dense tissues or images with overlapping cells. StarDist’s adaptability to both 2D and 3D data makes it highly versatile for a range of imaging modalities. Mesmer incorporates pre-trained convolutional neural networks (CNNs) optimized for multiplexed tissue images. It seamlessly segments both nuclei and cytoplasmic compartments, ensuring robust performance across diverse tissue types without the need for manual annotations. Mesmer’s pre-training on extensive datasets allows it to generalize effectively, reducing the need for user intervention and overcoming limitations posed by traditional algorithms. Similarly, Cellpose introduces a generalist deep learning framework capable of segmenting cells with varied shapes, sizes, and densities. Unlike many task-specific algorithms, Cellpose employs a flow-based representation to predict directional flows of pixels toward cell centers, enabling accurate boundary delineation. Its ability to handle highly heterogeneous datasets, including images from fluorescent, brightfield, and phase contrast microscopy, makes it particularly powerful for real-world applications where cell morphology is highly variable.

The adoption of these deep learning models has significantly improved segmentation accuracy in challenging tissue microenvironments, surpassing traditional and machine learning-based methods. By automating feature extraction and learning complex relationships in image data, these models not only streamline the segmentation workflow but also enhance the precision of downstream analyses, such as cell phenotyping and spatial analysis. These advances are crucial for extracting biologically relevant insights from high-dimensional imaging datasets in biomedical research.

### Cell phenotyping

Advancements in computational methods have significantly refined cell phenotyping. Clustering techniques, for instance, have become essential tools for grouping cells based on molecular profiles. One prominent method, PhenoGraph (56), constructs a weighted graph of cellular neighborhoods by identifying k-nearest neighbors in expression space and quantifying overlap using the Jaccard similarity coefficient (57). The graph is then partitioned into clusters, enabling the detection of subtle subpopulations in tissues. This approach has been widely applied to imaging datasets such as IMC, offering a robust framework for clustering. Another impactful technique, FlowSOM (58), utilizes self-organizing maps (SOMs) (59) for dimensionality reduction and clustering. It incorporates meta-clustering via minimal spanning trees, achieving results that are not only highly accurate but also orders of magnitude faster than traditional algorithms like SPADE (Sequential PAttern Discovery using Equivalence classes) (60). These methods provide scalable alternatives to manual gating, especially for high-dimensional dataset. Notably, several studies on melanoma (24, 33) have leveraged PhenoGraph, either alone or in combination with FlowSOM, for cell type identification, highlighting the power of these algorithms in unraveling tumor heterogeneity.

Machine learning further enhances phenotyping by incorporating prior biological knowledge and leveraging spatial context. Garnett (61), for example, is an interpretable framework that enables the rapid annotation of cells across tissues and species, even supporting hierarchical classification of subtypes. Garnett does not require prior clustering, making it adaptable to various datasets. CELESTA (62) expands on this approach by integrating spatial information into the classification process. It assigns cell types to “anchor cells” based on marker profiles and refines the phenotyping of ambiguous “non-anchor cells” using spatial relationships with neighboring cells. By employing probabilistic models and spatial scoring functions, CELESTA excels in classifying cells with uncertain identities. Astir (63), instead, takes a complementary approach by using deep recognition neural networks to assign probabilistic cell type identities from predefined marker sets. It is especially effective in scaling to massive datasets, delivering results with remarkable speed and precision.

Deep learning methods have revolutionized phenotyping by exploiting the detailed spatial and molecular features of multiplexed imaging data. For example, DeepCellTypes (64) combines visual encoders, language encoders, and channel-wise transformers to generalize across diverse datasets, seamlessly adapting to different imaging modalities and marker panels. CellSighter (65), another deep-learning-based pipeline, employs convolutional neural networks to classify cells probabilistically across imaging platforms, achieving inter-observer-level concordance in accuracy. Going further, STELLAR (SpaTial cELl LeARning) (66) utilizes geometric deep learning to analyze spatially resolved single-cell datasets. It integrates spatial and molecular features through graph convolutional neural networks, identifying known cell types from annotated reference datasets and discovering novel phenotypes in unannotated datasets. By leveraging spatial proximity and molecular expression, STELLAR provides a powerful tool for cell-type discovery and tissue structure analysis.

Collectively, these advanced methods have propelled cell phenotyping to new levels of accuracy and efficiency. They enable researchers to unravel the complexity of tissue organization and cellular interactions, seamlessly integrating phenotyping with spatial analysis to uncover deeper biological insights.

### Spatial analysis

Spatial analysis plays a pivotal role in uncovering the intricate organization of tissues and the dynamics of cellular interactions in multiplex imaging datasets. Building upon the foundational steps of preprocessing, segmentation, and cell phenotyping, it provides profound insights into tissue architecture and intercellular communication. Techniques such as neighborhood analysis, cellular community detection, and interaction modeling are particularly valuable in this context.

Neighborhood analysis focuses on understanding how different cell types are spatially distributed and interact within the tissue microenvironment. By evaluating spatial proximity, this approach can reveal critical insights into phenomena like immune infiltrates in tumors or the relationships between stromal and epithelial cells (67). Tools like HistoCAT (Histology Topography Cytometry Analysis Toolbox) (68) provide an accessible interface for exploring these spatial relationships in IMC data, allowing researchers to visualize cell phenotypes and compute interaction maps. The practical feasibility of HistoCAT has been demonstrated in clinical research: for instance, it was used by Xiao et al. (31) to identify spatially defined tumor-immune microenvironments associated with response to anti-PD-1 therapy in melanoma patients, and to classify distinct TME archetypes predictive of treatment outcome. Similarly, Martinez-Morilla et al. (30) employed IMC for biomarker discovery in metastatic melanoma, complementing spatial data analysis with quantitative methods to identify predictive markers such as beta2-microglobulin (B2M), supporting the potential of spatial proteomics for clinical stratification. Furthermore, Cytomapper (69), an R/Bioconductor package, offers powerful visualization capabilities for highly multiplexed imaging data, enabling researchers to generate informative spatial maps and explore cellular neighborhoods in detail, complementing the analytical strengths of HistoCAT. Meanwhile, R-based tools such as imcRtools (16) offer deeper statistical capabilities for calculating interaction probabilities and visualizing spatial patterns. This framework can be extended to analyze more general spatial patterns using packages like Spatstat (70) for advanced statistical assessments of spatial clustering and randomness.

Detecting cellular communities adds another layer of complexity by identifying clusters of cells that form functional units, such as immune niches or tumor microenvironments. Leveraging the same robust framework provided by imcRtools, researchers can cluster and characterize cellular communities in IMC data, integrating spatial metrics with phenotypic profiles. Additionally, software like Squidpy (71), which utilizes spatial neighborhood graphs, enables classification of cells into communities while incorporating multi-omic data such as gene or protein expression. These analyses can highlight patterns like immune deserts or coordinated interactions between stromal and immune cells, with Squidpy’s visualization tools offering an intuitive way to explore these relationships. The feasibility of Squidpy-based pipelines in clinical research is illustrated by Coullomb et al. (72), who developed MOSNA, a spatial omics analysis framework compatible with Squidpy, to uncover spatial features predictive of immunotherapy response and survival across cancer cohorts. By integrating spatial proteomics data with clinical metadata, their study demonstrates how cell interaction patterns and tissue architecture can inform patient stratification and treatment outcomes.

Interaction analysis delves deeper into the mechanisms of cell-cell communication by quantifying and modeling direct or indirect interactions. This is crucial for understanding how cells influence each other’s functions within their spatial context. imcRtools facilitates the computation of interaction frequencies and enrichment scores to identify preferential or avoided interactions between cell types (73). Advanced tools like Giotto (74) complement these efforts by detecting spatial dependencies that extend beyond mere proximity, helping to elucidate the spatial organization of functional phenotypes.

An important resource for the scientific community is Aquila (75), a spatial omics database and analysis platform that aims to centralize data, analysis tools, and visualizations, facilitating sharing and discovery in this rapidly growing field. This database could be invaluable for melanoma researchers seeking publicly available spatial proteomics datasets and tools for comparative analyses.

As spatial analysis techniques continue to evolve, integrative approaches are emerging that bridge spatial organization with molecular communication. Tools such as CellChat (76) and SpaOTsc (Spatial Optimal Transport for single-cell transcriptomics data) (77) model ligand-receptor signaling networks, offering a functional perspective on cell-cell interactions (78) These advancements not only enhance our understanding of tissue architecture but also open new avenues for exploring pathological processes.

Looking ahead, the field is poised for innovations that will improve scalability and interoperability across platforms, enabling researchers to tackle increasingly large and complex datasets. By synthesizing spatial metrics with molecular data, future studies promise to unveil deeper insights into the interplay between spatial organization and tissue functionality.

## Data integration for spatial analysis

### Integration of spatial proteomics with other tissue imaging approaches

While single imaging modalities can yield valuable information, integrating data across multiple platforms enables a more holistic view of the tissue microenvironment. This approach introduces challenges such as spatial misalignment (79) due to differences in platform resolution, data normalization, and the choice of integration methodologies.

Among integrative approaches, combining IF with hematoxylin and eosin (H&E) staining represents a straightforward but effective strategy (80). While H&E provides fundamental morphological information such as cell shapes, sizes, and tissue organization, IF enables visualization of multiple fluorescent markers within individual cells, offering molecular and structural insights. Tools like the Orion (81) or HIPI (H&E Image Interpretation and Protein Expression Inference) platform (82) take this integration further by seamlessly combining multiplex fluorescence with histological data, facilitating a comprehensive understanding of both cellular and tissue-level features.

The integration of IF with IMC (83, 84) exemplifies a more advanced approach, leveraging the strengths of both modalities while mitigating their individual limitations. By integrating IF and IMC, researchers can align high-resolution imaging capabilities with comprehensive molecular profiling, creating a synergistic workflow. This integration addresses the limitations of each technique: IF compensates for IMC lower resolution, while IMC extends IF multiplexing capacity. Computational advances, including multimodal image co-registration and machine learning, now enable pixel-level alignment of IF and IMC datasets, linking cellular phenotypes with molecular signatures in unprecedented details.

These integrative strategies have been transformative in practical applications. In tumor microenvironment studies, IF provides precise mapping of immune-tumor boundaries and structural features like vascular networks, while IMC captures phenotypic diversity and functional pathways in immune subsets and stromal compartments. This dual-layer analysis not only enhances our understanding of spatially resolved phenotypes but also identifies potential therapeutic targets by linking molecular mechanisms to tissue architecture.

Despite their transformative potential, integrating multiplex platforms introduces challenges, including data complexity, the need for standardized workflows, and alignment of modalities with varying spatial resolutions. However, ongoing advancements in computational tools and reproducibility standards are addressing these hurdles, ensuring that integrative approaches are both scalable and reproducible. As a result, the integration of multiplex imaging technologies is becoming a cornerstone of spatial biology, unlocking unprecedented insights into tissue architecture, cellular interactions, and functional diversity.

### Integration of spatial proteomics with other multi-omic approaches

Multi-omic data integration leverages computational advancements to analyze individual biomolecules within single cells. Each omics technique, such as RNA-seq, DNA methylation, and metabolite profiling, provides deeper insights into cellular interactions within their environment. However, each omics approach focuses on different aspects of cellular identity, with distinct strengths and weaknesses. Multi-omics integration offers a powerful method for robust and sensitive cell type/state identification, enhancing our understanding of cellular differentiation, gene regulatory networks, cell-cell interactions, microenvironmental organization, cellular lineages, and clonal dynamics. Meaningful integration of high-dimensional data, however, requires the development of computational and statistical models that account for the technical and biological complexities of these technologies (85). Argelaguet et al. (86), recently categorized data integration strategies into three main categories based on the anchors used to link different data modalities. Horizontal integration relies on common data features measured across different datasets, such as integrating across batches or technologies measuring the same analyte. Vertical integration involves parallel measurements of non-overlapping data features within the same cells, while diagonal integration is used when neither cells nor common features are available to serve as anchors. Although multimodal integration is advancing biomedical research, its clinical application is still in the early stages. Challenges include the high costs of multi-omics technologies, the need for specialized computational tools, and the requirement for rigorous clinical validation, which can be time-consuming and expensive. Despite these obstacles, multimodal integration holds great promise for enhancing our understanding of complex diseases and improving patient care in the future (87).

## Spatial proteomics: limitations and future outlook

Spatial proteomics has significantly advanced our understanding of melanoma TME by providing detailed, spatially resolved maps of the complex interactions between tumor cells, immune cells, and stromal components. These approaches have illuminated how cell-to-cell interactions influence key aspects of cancer biology, including disease progression, metastatization, and therapeutic response.

In melanoma, spatial proteomics has refined our understanding of the relationship between ICIs responses and the conventional pathology assessments of PD-1/PD-L1 expression, as well as the prognostic significance of “brisk” versus “non-brisk” immune infiltration. By identifying spatial patterns within the TME and characterizing specific cellular functional states, these techniques have elucidated the TME evolution during disease progression and identified elements associated with increased invasiveness and response to immunotherapies. Furthermore, mapping the spatial context of tumor-infiltrating immune cells, their interactions with tumor cells, and the expression of key immune checkpoint markers has advanced the concept of precision oncology.

As spatial proteomics continues to evolve, it is gradually emerging as new tool to be integrated into clinical practice, holding transformative potential for pathology and its application in cancer diagnosis, prognostication, and treatment.

Studies in melanoma have demonstrated the potential of spatial proteomics to inform clinical decision-making across various aspects of melanoma management, including: identifying prognostic biomarkers of survival (22, 23, 28, 31, 34, 37, 39); predicting response to immunotherapy (19, 27, 30, 32, 35, 36), characterizing patterns of disease progression (24, 25, 28); and uncovering novel mechanisms to optimize future immunotherapies (29, 33, 38).

Despite the recent advances, spatial proteomics has several limitations, and further improvements are still needed (88).

All the multiplex approaches developed so far offer cellular maps of the TME, however, they often overlook key factors such as the extracellular matrix (ECM) and soluble molecules (e.g. cytokines, chemokines, and metabolites) that directly influence the formation of specific cellular neighborhoods within the TME. New technologies, such as Deep Visual Proteomics (DVP), are addressing these limitations by combining the strengths of digital pathology with high-sensitivity mass spectrometry (MS). DVP enables the selective capture of cells for in-depth analysis, facilitating a more comprehensive comparison of relevant cellular states at higher throughput. Unlike traditional methods, DVP is not limited by antibody availability, allowing for the quantification of up to 10,000 proteins. Additionally, super-resolution protein imaging in DVP allows for detailed examination of protein localization at the subcellular level, providing insights into how proteins function in both health and disease. In melanoma, the application of DVP to classify cell states based on proteomic profiles has uncovered spatial proteome changes that occur during melanoma progression (89). This capability could uncover new therapeutic targets, advancing drug discovery and treatment strategies (90).

Tissues are inherently heterogeneous, and most of the spatial proteomic approaches have largely relied on the analysis of 2D specimens from single tissue slices. To better capture the complexity and diversity of tissues, various research groups are working to develop 3D spatial proteomic workflows. Most of them are based on sequential slide staining coupled with tissue reconstruction (91) or on tissue clearing protocols combined with multiplex staining (92, 93).

Another limitation of spatial proteomics is the large volume of data it generates, which often requires several days for thorough analysis. The development of rapid, automated data storage platforms and more efficient analysis pipelines could greatly accelerate data processing, enabling faster insights and enhancing the overall utility of spatial proteomics in research and clinical applications. This advancement would not only accelerate results but also pave the way for the clinical application of spatial proteomics, potentially revolutionizing pathology.

The integration of artificial intelligence (AI) with multiplex imaging, traditional digital pathology approaches (94), and spatial analysis represents a transformative opportunity for spatial proteomics.

AI-driven approaches, particularly in computer vision, could revolutionize how we analyze complex datasets, enabling real-time pattern recognition and spatial mapping that surpass human capabilities. These techniques could also refine traditional digital pathology by enhancing the consistency and resolution of image analysis, enabling more detailed tissue characterization. AI could interpret the intricate spatial relationships within the tumor microenvironment, identifying biomarkers and predicting responses to immunotherapies with unparalleled precision (95). Emerging tools (46), promise to unify tasks like segmentation, classification, and phenotyping into streamlined pipelines, significantly enhancing both the accuracy and efficiency of spatial data analysis (96). Looking forward, AI-powered platforms could offer intuitive, query-based interfaces for integrating imaging data with clinical and molecular profiles, paving the way for new biological insights and personalized treatment strategies. Such innovations would not only accelerate data processing but also address the inherent limitations of human subjectivity, unlocking the full potential of multiplexed spatial proteomics in research and clinical practice.

There is a significant global effort to create comprehensive human atlases of cell networks and neighborhoods, spanning a wide range of tissue types and disease states. These atlases aim to map the intricate cellular interactions and spatial organization within different tissues, providing a rich resource for understanding human biology in health and disease (97–99).

In addition, recognizing the systemic nature of cancer, where tumor-induced perturbations extend beyond the local TME, future spatial proteomic studies should be integrated with analyses of cancer-mediated changes occurring at the systemic level, such as those observed in peripheral blood. This integrated approach could help to identify soluble factor signatures (e.g. metabolites, proteins) indicative of the TME that are amenable to detection in liquid biopsies.

To conclude, the integration of spatial multi-omics represents a frontier in biomedical research, offering unprecedented opportunities to uncover the spatial and functional complexity of biological systems. Future efforts will likely focus on refining computational frameworks, reducing technological costs, and bridging the gap toward clinical applications. By addressing these challenges, spatial multi-omics could transform our understanding of tissue organization and disease mechanisms, paving the way for personalized diagnostics and therapeutics.
